# Enhanced Porcine Reproductive and Respiratory Syndrome Virus Replication in Nsp4- or Nsp2-Overexpressed Marc-145 Cell Lines

**DOI:** 10.3390/vetsci12010052

**Published:** 2025-01-13

**Authors:** Zhengqin Ye, Zhenbang Zhu, Liangzheng Yu, Zhendong Zhang, Xiangdong Li

**Affiliations:** 1 Jiangsu Co-Innovation Center for Prevention and Control of Important Animal Infectious Diseases and Zoonoses, College of Veterinary Medicine, Yangzhou University, Yangzhou 225012, China; dz120220017@stu.yzu.edu.cn (Z.Y.); zhuzhb@yzu.edu.cn (Z.Z.); dz120210020@stu.yzu.edu.cn (L.Y.); 008686@yzu.edu.cn (Z.Z.); 2 Joint International Research Laboratory of Agriculture and Agri-Product Safety, Ministry of Education of China, Yangzhou University, Yangzhou 225012, China

**Keywords:** PRRSV, Nsp2, Nsp4, Marc-145 cell line, replication

## Abstract

Porcine reproductive and respiratory syndrome (PRRS) causes significant economic losses to the swine industry. The killed PRRSV vaccine has been reported to be safe and could elicit humoral responses. The killed PRRSV vaccine with a high viral antigen load combined with robust adjuvants could provide good protection against the infection. However, the high virus titer of PRRSV on the successive production cell lines is the prerequisite for this strategy. In this study, we explored PRRSV production in two recombinant Marc-145 cell lines expressing Nsp2 or Nsp4 through a lentivirus system. The results demonstrated that either Nsp2 or Nsp4 expressing Marc-145 cells did not affect cell morphology and growth kinetics but significantly enhanced PRRSV replication. Overall, our exploration may enable the production of high-yield PRRSV and offer a potential tool for developing safer and more effective PRRSV vaccines.

## Simple Summary

The porcine reproductive and respiratory syndrome virus (PRRSV) is a major threat to pig farms globally, causing significant economic losses. The killed PRRSV vaccines with a high load of antigens might help induce humoral immune responses. In the present study, Marc-145 cell lines overexpressing viral Nsp4 or Nsp2 were constructed, and both engineered cell lines significantly enhanced virus production, particularly at the early stages of infection. Our results provide a promising strategy for developing safe and effective PRRSV vaccines by enabling the production of a large quantity of the virus economically.

## 1. Introduction

Porcine reproductive and respiratory syndrome (PRRS) poses a significant global threat to the swine industry, resulting in substantial economic losses [1,2]. It is caused by the PRRS virus (PRRSV) and is recognized as reproductive failure of sows and respiratory problems of piglets and growing pigs [3]. Taxonomically, there are two types of PRRSV, PRRSV type 1 and type 2. According to the newest classification, previous PRRSVs are classified into two distinct species named *Betaarterivirus suid 1* and *Betaarterivirus suid 2*, classified within two different subgenera. Effective prevention and control of PRRS remains a critical challenge for the global swine industry. Although biosecurity has been confirmed as a primary strategy, most producers have adopted vaccination strategies [4,5]. Modified-live PRRSV vaccines (MLV) can induce a protective immune response against homologous viruses. However, MLVs can replicate in the host, increasing the risk of reversion to virulence, and making them ‘leaky’ vaccines [6,7]. Compared to MLV, the killed PRRSV vaccine (KV) is safer, but the KV-induced protective immunity mediated by humoral immune responses may not be satisfactory [8,9,10]. In recent years, multiple novel strategies and techniques have been explored to focus on developing killed PRRSV vaccines in China, such as the use of novel adjuvants and protein-expressing systems targeting potential specific antigens [11,12]. Due to the uncertainty of available protective viral antigens, the killed whole PRRS virus with a high antigen load is the primary choice economically, which prompts the development of an effective killed PRRSV vaccine by producing a high-titer virus.

PRRSV non-structural protein 2 (Nsp2) plays a multifaceted role in the viral life cycle, including antagonizing host antiviral responses [13,14,15], an especially functional role in facilitating the assembly of the N protein with viral envelope proteins in the PRRSV life cycle [16,17]. Previous studies have demonstrated that cells expressing the Nsp2 exhibit accelerated PRRSV replication, particularly during the early stages of infection (24–48 hpi) [15], suggesting the potential of developing cell lines specifically engineered to enhance PRRSV proliferation. Non-structural protein 4 (Nsp4), a 3C serine protease family member, possesses cleavage enzyme activity and is responsible for the processing of Nsp3–Nsp12, key roles in both the life cycle and virulence of the PRRSV [18], which cunningly evades the host’s immune defenses by targeting multiple critical pathways. It disrupts key signaling cascades, directly inhibits essential transcription factors, and blocks the production of type I interferon, effectively preventing the host from mounting an effective antiviral response and allowing the virus to establish infection successfully [19,20,21,22,23]. Besides, Nsp4 exhibits high conservation with minimal mutations, in contrast to the high variability of Nsp2 [24]. Therefore, we speculated that cells overexpressing Nsp4 could be viable and promote viral replication.

In the present study, we constructed the Nsp2- or Nsp4-overexpressed Marc-145 cell lines using a recombinant lentivirus transduction system and analyzed the effect on PRRSV replication.

## 2. Materials and Methods

### 2.1. Cells and Viruses

Marc-145 and HEK293T cells were preserved in our laboratory and cultured in Dulbecco’s Modified Eagle’s Medium (DMEM) (Corning, USA) supplemented with 10% fetal bovine serum (FBS) (Gibco, USA) at 37 °C in an atmosphere of 5% CO_2_ [25]. The use of the above two cell lines was approved by the Institutional Ethic Committee of Yangzhou University with permit No. 20240401. Three type 2 PRRSV strains (CHR6, SD1612-1, and XJ17-5) were used in this study. The CHR6 strain was preserved in our laboratory [25,26], and the SD1612-1 (GenBank: MN119304.1) and XJ17-5 (GenBank: MK759853.1) strains were generously provided by Prof. Nanhua Chen from Yangzhou University [25]. All PRRSV strains were propagated in Marc-145 cells and titrated as a 50% tissue culture infective dose (TCID_50_) according to previously established methods [15].

### 2.2. Construction of Nsp2 or Nsp4 Expression Plasmids

The open reading frames (ORFs) encoding PRRSV Nsp2 or Nsp4 were obtained from the PRRSV CHR6 strain. Reverse transcription polymerase chain reaction (RT-PCR) was performed to amplify the full-length *Nsp2* and *Nsp4* genes using the primer pairs *Nsp2*-LF/*Nsp2*-LR and *Nsp4*-LF/*Nsp4*-LR, respectively (underlined sequences indicate homology arms, see Table 1). Total RNA was extracted from PRRSV-infected cells for RT-PCR. Each PCR amplicon was individually inserted into a pCN179-*puro-GFP* lentiviral vector (You Bio, China) and sequenced.

### 2.3. Production of Recombinant Lentivirus

HEK293T cells were seeded in 35 mm dishes 20 h before transfection. A DNA mixture containing pCN179-*puro-GFP-Nsp2* (3 μg), pCN179*-puro-GFP-Nsp4* (3 μg) or pCN179-*puro-GFP* (3 μg), envelope plasmid pMD2.G (You Bio) (2 μg), and packaging plasmid pSPAX2 (You Bio) (1 μg) were diluted in 250 μL of serum-free MEM. Separately, 18 μL of polyethyleneimine (PEI) was diluted in 250 μL serum-free MEM. Both solutions were incubated for 5 min at room temperature. After incubation, the diluted plasmids were combined with diluted PEI, gently mixed, and incubated for an additional 20 min at room temperature. The resulting complexes were then added to each well. Cells were incubated at 37 °C in a CO_2_ incubator for 48 h. The medium was replaced with DMEM supplemented with 2% FBS after 6 h of incubation.

Supernatants were collected 48 h post-transfection, centrifuged at 2000 rpm for 10 min to remove cell debris, and filtered through a 0.22 μm filter. The filtered supernatant was concentrated on a 100 kDa Amicon Ultra (Millipore) device. The viral titer of the recombinant lentivirus was determined by end-point dilution assay using a 96-well plate with four replicates per dilution. The virus titer, expressed as 50% tissue-culture infectious dose (TCID_50_)/0.1 mL, was calculated using the Reed–Muench formula.

### 2.4. Construction of Marc-145 Cell Lines Overexpressing Nsp2 or Nsp4

To establish stable cell lines expressing Nsp2 or Nsp4, Marc-145 cells were seeded in 24-well plates at a density to achieve 40% confluency after 24 h of culture in DMEM supplemented with 10% FBS. Cells were then infected with recombinant lentiviruses (MOI = 10) carrying the *Nsp2* or *Nsp4* gene expression cassette. Four hours post-infection, the medium was replaced with fresh DMEM supplemented with 10% FBS. Selection for transgene expression was achieved by treating cells with 8 μg/mL puromycin (MCE, USA) for 72 h. After three rounds of selection, surviving cells were positively sorted for Nsp2 or Nsp4 expression.

### 2.5. Quantitative Real-Time PCR (qRT-PCR)

qRT-PCR assessed the transcription levels of *Nsp2* and *Nsp4* in the cell lines. Total RNA was extracted from cells using TRIzol reagent (TIANGEN, China). According to the manufacturer’s instructions, reverse transcription was performed using HiScript III-RT SuperMix (Vazyme, China). qRT-PCR was performed using a ChamQ Universal SYBR qPCR Master Mix (Vazyme, China) on QuantStudio3 (Applied Biosystems). Reverse-transcribed products were used as templates to amplify the indicated genes with primers listed in Table 1. Data were normalized to *GAPDH* in each sample [25]. Relative mRNA expression was calculated using the 2^−∆∆Ct^ method, with three replicates included for each treatment.

### 2.6. Cell Proliferation Analysis

Cell proliferation was examined by a cell growth curve assay [27]. Cells were plated onto six-well plates at a density of 20,000 cells per well, and growth curves were generated from cell counts over six consecutive days.

### 2.7. Effects of Nsp2 or Nsp4 Expression on PRRSV Replication

To determine the effect of PRRSV Nsp2 or Nsp4 on viral replication, the growth kinetics of PRRSV CHR6 were examined in r*Nsp2*-Marc-145 and r*Nsp4*-Marc-145 cells infected at an MOI of 1 [25]. Viral titers were determined at 12, 24, 36, 48, and 60 hpi using the method described in the reference [15]. PRRSV replication in Marc-145 cells was also assessed by immunofluorescence assay (IFA) and Western blot [25,28]. Membranes were incubated with primary antibodies, including anti-PRRSV N (MEDIAN, Republic of Korea), anti-GAPDH, anti-GFP (Cell Signaling Technology, USA), mouse mAbs specific for PRRSV Nsp2 (provided by Dr. Yandong Tang from Harbin Veterinary Research Institute of Chinese Academy of Agricultural Sciences [16]), and mouse mAbs specific for PRRSV Nsp4 (provided by Dr. Yihong Xiao from Shandong Agricultural University [29]). Three replicates were included for each treatment.

### 2.8. Statistical Analyses

Statistical analysis was performed using GraphPad Prism 9. Data are presented as mean ± standard deviation (SD). Two-group comparisons were conducted using an unpaired Student’s *t*-test with unequal variances. *p* < 0.05 was considered to be significant.

## 3. Results

### 3.1. Construction of Marc-145 Cell Lines Overexpressing Nsp2 or Nsp4

**Figure 1:**
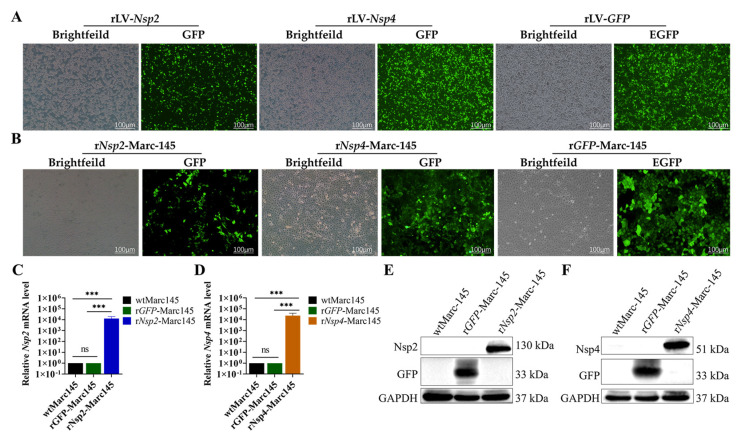
Construction of stable transfected Marc-45 cell clones. (**A**) Generation of rLv-*Nsp2* or rLv-*Nsp4* lentiviruses. HEK293T cells transfected with pCN179-*puro-Nsp2* or pCN179-*puro-Nsp4* plasmid and packaging, envelope plasmids at 48 h post-transfection. Brightfield and GFP fluorescence images are shown. Scale bar = 100 µm. rLV-*GFP* serves as a negative control. (**B**) Single Marc-145 cells 5 days after puromycin selection. While several cells without GFP expression survived, only cell clones with GFP expression, indicative of successful lentiviral infection, were selected. Brightfield and GFP fluorescence. (**C**) Endogenous *Nsp2* mRNA levels were examined by real-time qRT-PCR. GAPDH was used as an internal control. Data are the results of three independent experiments (means ± SD). Data are the results of three independent experiments (means ± SD). Significant differences are denoted by * *p* < 0.05, ** *p* < 0.01, and *** *p* < 0.001. (**D**) Endogenous *Nsp4* mRNA levels were examined by real-time qRT-PCR. GAPDH was used as an internal control. Data are the results of three independent experiments (means ± SD). Data are the results of three independent experiments (means ± SD). Significant differences are denoted by * *p* < 0.05, ** *p* < 0.01, and *** *p* < 0.001. (**E**) Detection of viral protein expression by Western blotting using Nsp2 monoclonal antibody as the primary antibody in r*Nsp2*-Marc-145 cells. The r*GFP*-Marc-145 cells were shown as a control. (**F**) Detection of protein expression by Western blotting using Nsp4 monoclonal antibody as the primary antibody in r*Nsp4*-Marc-145 cells. The r*GFP*-Marc-145 cells were shown as negative control.

pCN179-*puro-GFP-Nsp2*, pCN179-*puro-GFP-Nsp4*, and pCN179-*puro-GFP* plasmids and packaging and envelope plasmids were co-transfected into HEK293T cells to acquire recombinant lentiviruses, respectively. After 24 h post-transfection, green fluorescence was detectable, and approximately 90% GFP expression was observed after 48 h in both groups (Figure 1A). Recombinant viruses were harvested and concentrated, yielding a viral titer of 10^6.0^ TCID_50_/0.1mL for the Nsp2-expressing virus and 10^6.75^ TCID_50_/0.1 mL for the Nsp4-expressing virus. Then, Marc-145 cells were transduced with lentivirus expressing either Nsp2, Nsp4, or GFP with MOI = 10. As shown in Figure 1B, successful transduction was confirmed by green fluorescence detection 24 h post-transduction. Following three rounds of puromycin selection to eliminate uninfected cells, the Nsp2, Nsp4, or GFP overexpressed Marc-145 cell lines were successfully constructed (Figure 1C–F). The original WB images were available in the Supplementary File.

### 3.2. Nsp2 or Nsp4 Expression Has No Effect on Cell Morphology or Growth

**Figure 2:**
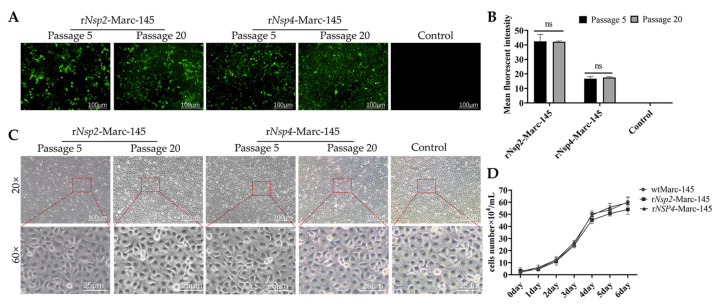
The expression of Nsp2 or Nsp4 does not impact cell morphology or growth. (**A**) r*Nsp2*-Marc-145 cells or r*Nsp4*-Marc-145 cells that were GFP-positive at low passage (P5) and high passage (P20) under fluorescence microscopy. Magnification: 100×. Scale bar = 20 µm. (**B**) Quantification of GFP-positive cells by ImageJ software. (**C**) Compare the morphology of r*Nsp4*-Marc-145 cells or r*Nsp2*-Marc-145 cells with wtMarc-145 at low passage (P5) and high passage (P20) under phase contrast microscopy and local magnification. Scale bar = 100 µm. (**D**) Growth kinetics of Nsp2- or Nsp4-expressing cell lines. The 6-well tissue culture plates were individually seeded with 2 × 10^4^ cells/mL of the engineered cells or parental cell line (Marc-145). Cell growth was monitored every 24 h over 6 days.

The Nsp4 or Nsp2 expression stability in Marc-145 cells was evaluated over 20 passages, respectively. Protein expression was assessed using fluorescence microscopy (Figure 2A) and quantified by ImageJ software (Figure 2B), revealing that Nsp2 or Nsp4 expression remained remarkably stable throughout the experiment, with no significant variations compared to the initial passage. Besides, the morphology of both r*Nsp2*-Marc-145 cells and r*Nsp4*-Marc-145 cells growing in T75 culture flasks remained remarkably consistent over the passage range P5-P20 (Figure 2C). Further, the growth of cells expressing Nsp4 or Nsp2 was evaluated by comparing them with the non-recombinant parental line. The overall growth kinetics of each Nsp4- and Nsp2-expressing cell line were comparable to those of the parental Marc-145 cells (Figure 2D). The above results suggest that the expression of Nsp4 or Nsp2 does not negatively impact the growth characteristics of the Marc-145 cells.

### 3.3. Overexpressed Nsp2 or Nsp4 Marc-145 Cell Lines Enhance PRRSV Production

**Figure 3:**
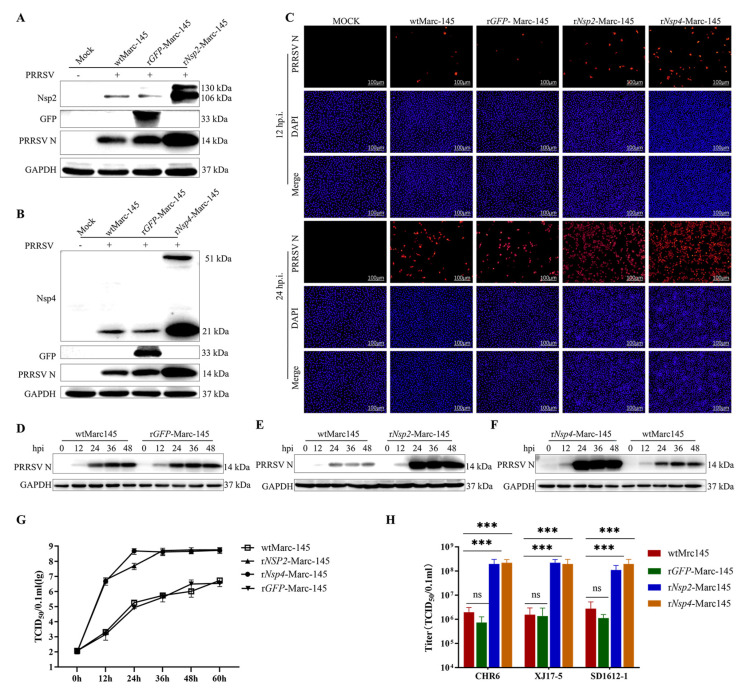
Overexpressed Nsp2 or Nsp4 in Marc-145 cell lines facilitate PRRSV production. (**A**) Western blot analysis of viral N protein levels in the infected r*Nsp2*-Marc-145 cells at 24 hpi. Cells were mock infected or infected with PRRSV (MOI = 1). GAPDH was shown as an internal control. r*GFP*-Marc-145 cells were shown as a control. (**B**) Western blot analysis of viral N protein levels in the infected r*Nsp2*-Marc-145 cells at 24 hpi. Cells were mock infected or infected with PRRSV (MOI = 1). GAPDH was shown as an internal control. The r*GFP*-Marc-145 cells were shown as a control. (**C**) Immunofluorescence assay (IFA) analysis of PRRSV N protein expression in Marc-145 cells at 12 and 24 hpi. Cells were mock infected or infected with PRRSV (MOI = 1) and stained with a monoclonal antibody (mAb) recognizing the viral nucleocapsid N protein (red), and nuclei were counterstained with DAPI (blue). r*GFP*-Marc-145 cells were shown as a control. Scale bar = 100 µm. (**D**) Western blot analysis of viral N protein levels in the infected r*GFP*-Marc-145 cells at different time points (12, 24, 36, and 48 hpi). Cells were mock infected or infected with PRRSV (MOI = 1). GAPDH was shown as an internal control. (**E**) Western blot analysis of viral N protein levels in the infected r*Nsp2*-Marc-145 cells at different time points (12, 24, 36, and 48 hpi). Cells were mock infected or infected with PRRSV (MOI = 1). GAPDH was shown as an internal control. (**F**) Western blot analysis of viral N protein levels in infected r*Nsp4*-Marc-145 cells at different time points (12, 24, 36, and 48 hpi). Cells were mock infected or infected with PRRSV (MOI = 1). GAPDH was shown as an internal control. (**G**) Growth kinetics of PRRSV CHR6 in wtMarc-145, r*Nsp2*-Marc-145, and r*Nsp4*-Marc-145 cells. Cells were infected at an MOI of 1, and viral titers in the supernatants were determined using TCID_50_ assays at the indicated time points (0, 12, 24, 36, 48, and 60 hpi). r*GFP*-Marc-145 cells were shown as a control. Data were presented as mean ± SD (*n* = 3). (**H**) Viral yields in the Nsp2- and Nsp4-expressing cell lines infected with three strains (CHR6, XJ17-5, and SD1612-1). wtMarc-145, r*Nsp2*-Marc-145, and r*Nsp4*-Marc-145 cells were individually infected (MOI 1) with PRRSV strains CHR6, XJ17-5, and SD1612-1. At 60 hpi, progeny virus in the respective cell culture supernatants was quantified using the Reed–Muench method. r*GFP*-Marc-145 cells were shown as a control. These data represent three independent experiments performed in triplicate. Standard deviation bars are shown. A Student’s *T*-test with unequal variance was used to compare respective virus yields between each cell type and Marc-145 (* *p* < 0.05, ** *p* < 0.01, and *** *p* < 0.001).

To investigate the role of Nsp4 and Nsp2 in PRRSV replication and compare their effects to those of wild-type Marc-145 cells (wtMarc-145), PRRSV CHR6 replication was analyzed in both Nsp2- and Nsp4-expressing Marc-145 cell lines. Initial Western blot analysis, performed at 24 h post-infection (hpi), revealed that both Nsp2- and Nsp4-expressing cell lines significantly promoted PRRSV CHR6 replication (Figure 3A,B, WB blots were also given in Original Data of WB blots). Subsequently, immunofluorescence assay (IFA) and Western blot analysis were performed and revealed that both Nsp2- and Nsp4-expressing cell lines significantly promoted PRRSV CHR6 replication at different time points (Figure 3C–F, WB blots were also given in the Original Data of WB blots). The TCID_50_ of the virus in both recombinant cell lines ranged from 10^8.0^/0.1 mL to 10^8.5^/0.1 mL, significantly higher than the TCID_50_ of wtMarc-145 cells and r*GFP*-Marc-145 cells, which ranged from 10^5.75^/0.1 mL to 10^6.25^/0.1 mL, indicating significantly enhanced PRRSV growth in the recombinant cell lines (Figure 3G). Besides, viral yields in the Nsp2- or Nsp4-expressing cell lines infected with three different PRRSV strains (CHR6, XJ17-5, and SD1612-1) after 20 consecutive passages were consistently 100-fold higher than those observed in wtMarc-145 cells at 60 hpi, with no significant difference observed among these three strains (Figure 3H).

## 4. Discussion

Porcine reproductive and respiratory syndrome (PRRS) remains a significant economic burden on the global swine industry. The PRRSV strains in the field in China underwent substantial changes from classical PRRSV strains, highly pathogenic strains, and now the dominant NADC30-like PRRSV strains. At the same time, reversion and recombination between modified live vaccines (MLVs) and field viruses strongly exacerbate the complexity due to the wide use of MLVs [30,31]. Compared to MLVs, besides safer, inactivated PRRSV vaccines could produce neutralization antibodies specifically for homologous strains [32,33,34]. Recent research focuses on developing innovative PRRSV vaccines, exploring suitable adjuvants, nanotechnology-based delivery systems, and different inactivation processes to optimize vaccine efficacy and safety [35,36]. Methods like propiolactone (BPL) inactivation [37], UV-radiation [11], and binary ethylenimine (BEI) inactivation [38], often combined with adjuvants, have shown promise in increasing VN titers and reducing viremia following PRRSV challenge. Additionally, vaccination of pregnant sows with BEI-inactivated PRRSV has been demonstrated to reduce fetal lesions and viremia, although complete protection against congenital infection remains elusive. Nanoparticle-based vaccine delivery systems offer a promising approach due to their ability to enhance antigen uptake by immune cells, protect antigens from degradation, and promote cross-presentation, ultimately leading to stronger immune responses against infectious pathogens [35,38]. Studies using poly(lactide-co-glycolide) (PLGA) nanoparticles entrapping PRRSV antigens, alone or with adjuvants, have demonstrated reduced viremia, lung lesions, and enhanced immune responses, including increased virus-specific antibodies and interferon-gamma production [39]. Furthermore, Hyunil Kim et al. compared vaccine efficacy according to the virus antigen quantity and inactivation reagent and found that BEI-inactivated PRRSV vaccine with higher doses (10^6.0^ PFU/mL) has the potential boost neutralization antibody titers [40], suggesting higher PRRSV titers might hold the potential for improving inactivated vaccine efficacy.

Multiple strategies like viral adaptation and receptor overexpression have been explored to promote PRRSV replication [41,42,43]. Wang et al. found that JX143 was characterized by higher titers, faster growth kinetics, and larger plaque sizes as the passage number increased compared with the parental virus [43]. CD163, CD169, and CD151 have been identified as key receptors for PRRSV, and their overexpression has been shown to enhance PRRSV replication and increase viral titers. Recent studies have demonstrated that the BHK-21-TTG cell line, engineered to co-express CD163, CD169, and CD151, exhibited significantly higher viral loads compared to Marc-145 cells, indicating its enhanced susceptibility to PRRSV infection [42].

Nsp2 and Nsp4 are crucial for both the PRRSV life cycle [13,14,15,16,17,18] and the auto-proteolytic processing cascade that generates the remaining 14 non-structural proteins (NSPs), which themselves are also key proteases within the PRRSV life cycle [28]. In the present study, we investigated the potential of overexpressing PRRSV non-structural proteins to enhance viral replication. We successfully generated stable Nsp2- or Nsp4-overexpressing Marc-145 cell lines using a lentiviral three-plasmid system. Both cell lines exhibited robust growth characteristics and consistent expression over 20 passages. Importantly, infection with PRRSV CHR6 revealed that overexpression of either Nsp2 or Nsp4 significantly enhanced virus replication, resulting in a 100-fold increase compared to wild-type Marc-145 cells, and both cell lines exhibited similar susceptibility to different PRRSV strains infection. To ensure synchronous infection and minimize cytopathic effects during replication analysis, we used a 1 MOI. Preliminary data indicated that this MOI results in robust and measurable viral replication. Conversely, lower MOIs (<1) would lead to asynchronous infection, causing variations in infection timing and ultimately reducing overall viral replication.

Besides, we also analyzed the potential influence through overexpressing other conserved non-structural proteins, including Nsp6, Nsp8, and Nsp12 [24], but the results did not significantly enhance PRRSV replication, suggesting that Nsp2 and Nsp4 may play unique and crucial roles in PRRSV replication. The mechanisms by which Nsp2 and Nsp4 promote PRRSV replication, particularly their interaction with host factors and other viral proteins, are necessary to be studied in the future. Our exploration provides valuable insights into the roles of specific PRRSV non-structural proteins in enhancing viral replication. One limitation of our study is to evaluate the efficacy of PRRSVs produced in the above cell lines, which need to be tested on pigs in the future. While the r*Nsp2*-Marc-145 and r*Nsp4*-Marc-145 cell lines could be used for vaccine development and research, further investigation into their specific properties and applications is warranted.

## 5. Conclusions

In the present study, two Marc-145 cell lines overexpressing Nsp2 or Nsp4 were constructed, which showed an obvious positive role for PRRSV replication. Our results provide a promising strategy for developing safe and effective PRRSV vaccines.

## Figures and Tables

**Table 1:** Information of primers used in the study.

Primer	Sequence (5′-3′)
*Nps2*-LF	GAACCGTCAGATCCGCTAGTCCCGGGATGGCTGGAAAGAGAGCAAG
*Nps2*-LR	CCCTTGCTCACCATGGTGGCGGATCCGCCCAGTAACCTGCCAAGAATG
*Nps4*-LF	CGTCAGATCCGCTAGTCCCGGGATGGGCGCTTTCAGAACTCGAAA
*Nps4*-LR	TGCTCACCATGGTGGCGGATCCTTCCAGTTCGGGTTTGGCAG
*Nps2*-F	CTGTTTCGCAATTCTATG
*Nps2*-R	AGCAATCCTCAATAACTT
*Nps4*-F	TGTCCTTACGGGTAATTC
*Nps4*-R	GAGGATGTCAGCCAATAG
m*GAPDH*-F	TGACAACAGCCTCAAGATCG
m*GAPDH*-R	GTCTTCTGGGTGGCAGTGAT

## Data Availability

All Western blots presented in figures were given in the section of the Supplementary Materials which were named original data of WB blots. The data that support the findings of this study are available from the corresponding author, upon reasonable request.

## Supplementary Materials

The following supporting information can be downloaded at: https://www.mdpi.com/article/10.3390/vetsci12010052/s1.
