# Supplementary figures and images for: Enhanced Porcine Reproductive and Respiratory Syndrome Virus Replication in Nsp4- or Nsp2-Overexpressed Marc-145 Cell Lines
Source: Vet Sci. 2025 Jan 13;12(1):52. doi: 10.3390/vetsci12010052 (PMC11768971; doi:10.3390/vetsci12010052)

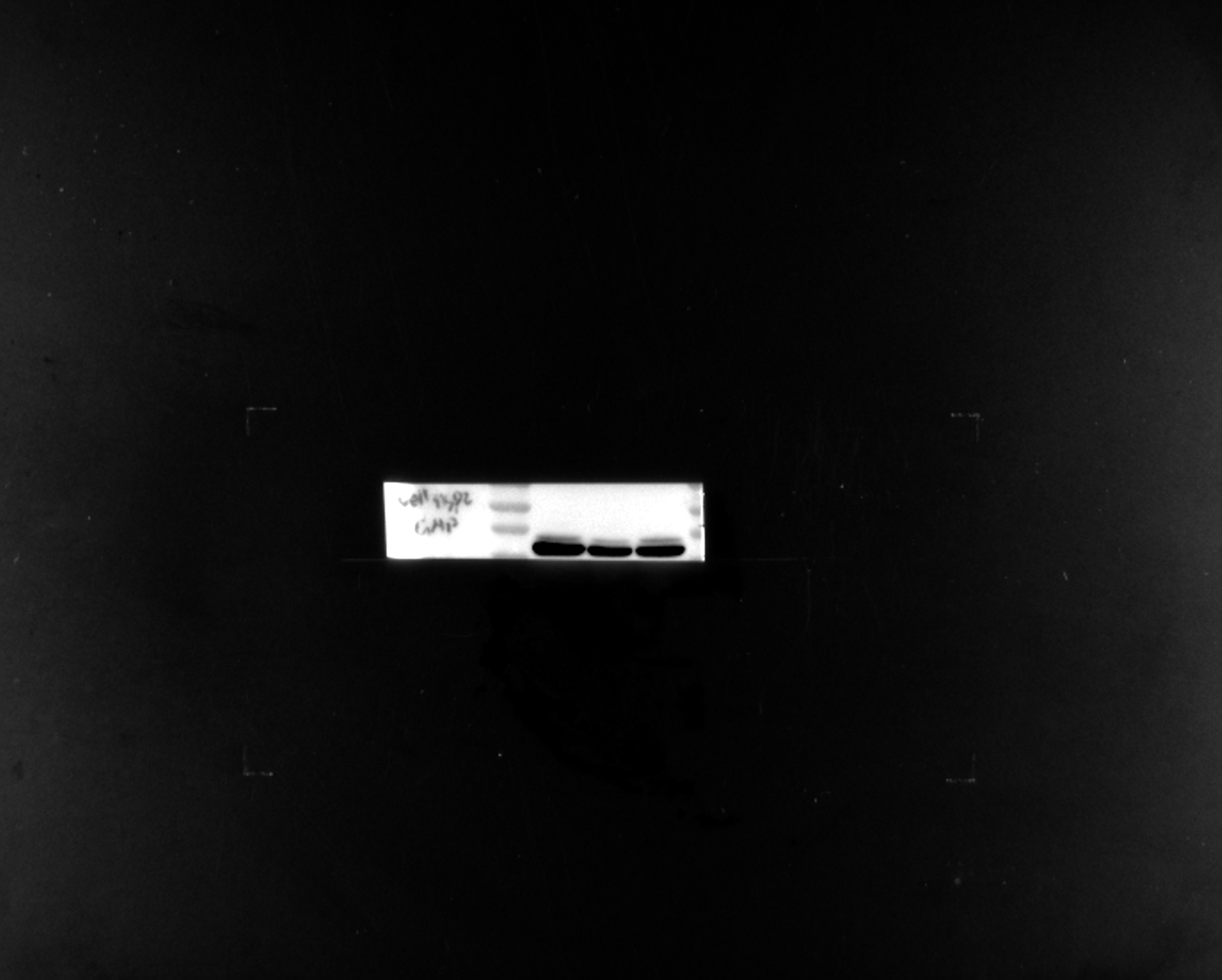

Supplement: Supplementary file 1 [file vetsci-12-00052-s001.zip › Gel原始图片/Fig 1E gel/Fig 1E GAPDH.Tif]

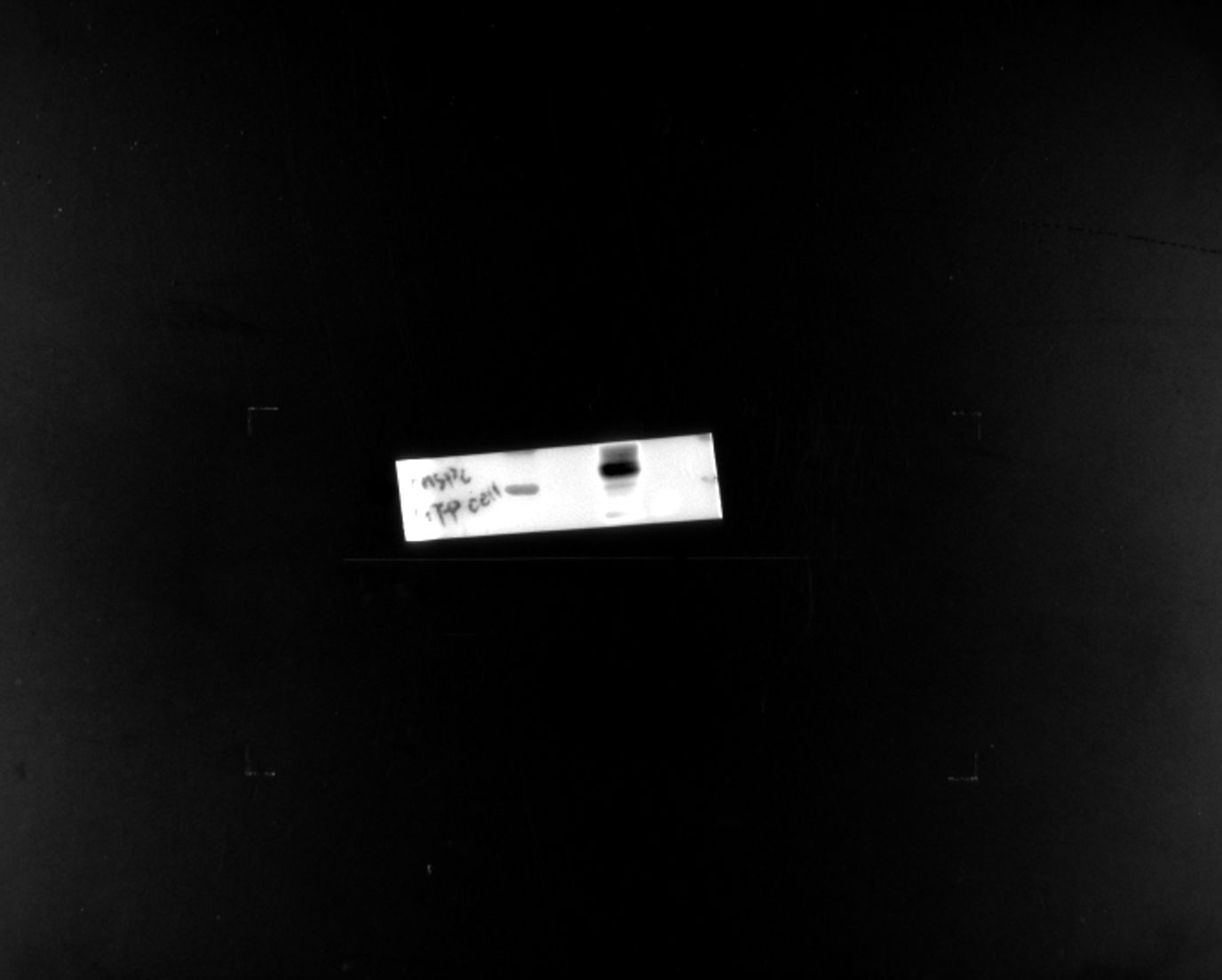

Supplement: Supplementary file 1 [file vetsci-12-00052-s001.zip › Gel原始图片/Fig 1E gel/Fig 1E GFP.Tif]

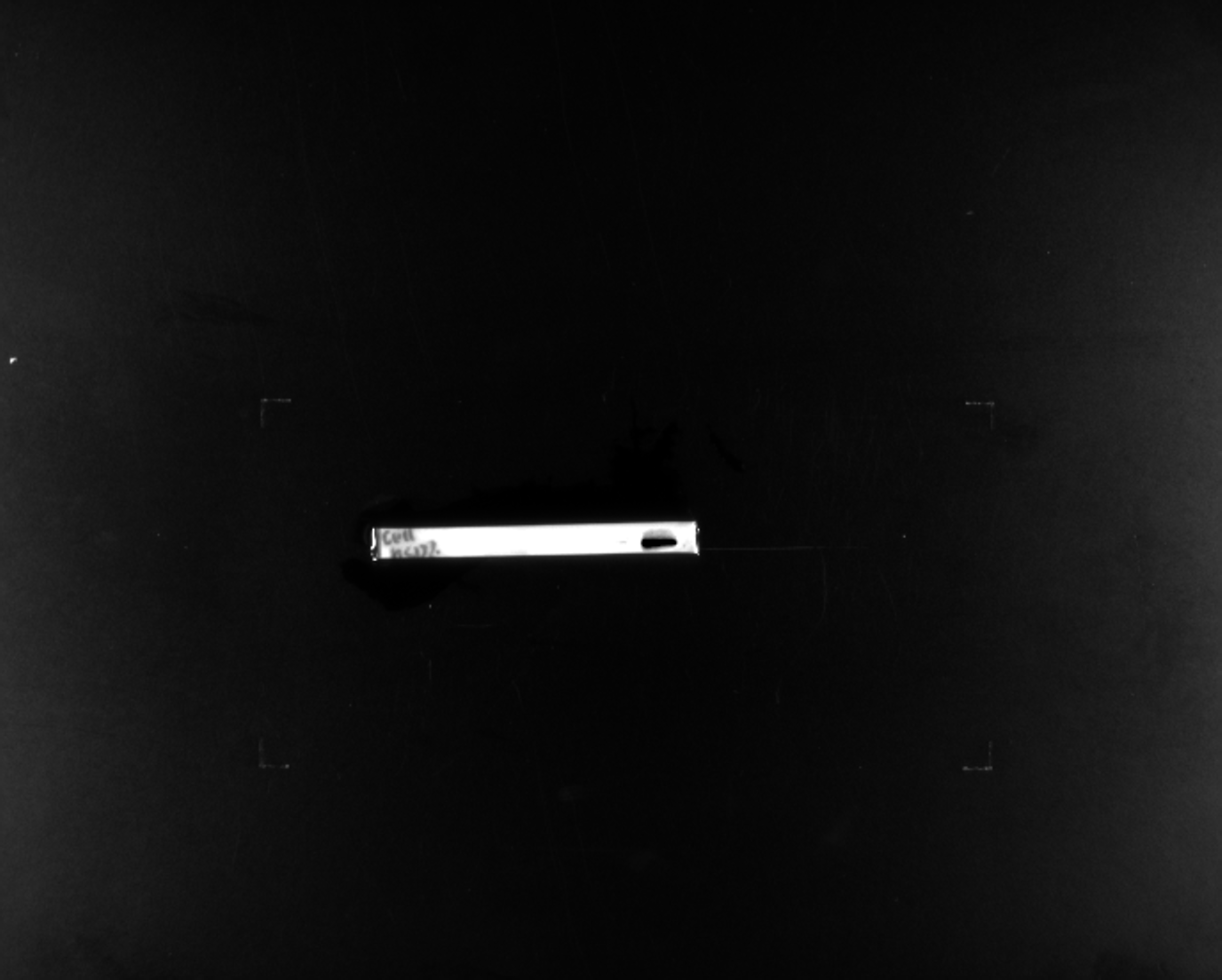

Supplement: Supplementary file 1 [file vetsci-12-00052-s001.zip › Gel原始图片/Fig 1E gel/Fig1E Nsp2.Tif]

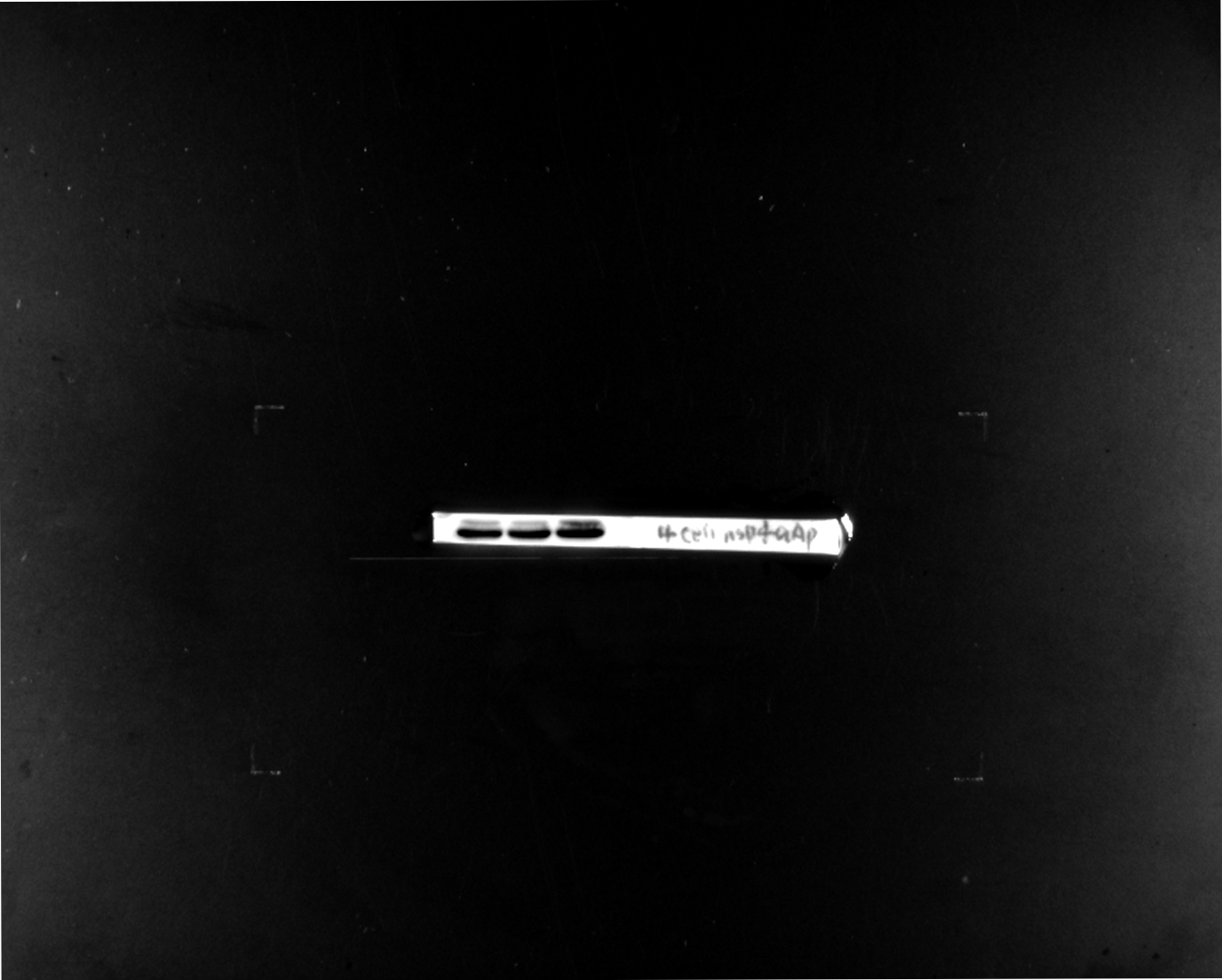

Supplement: Supplementary file 1 [file vetsci-12-00052-s001.zip › Gel原始图片/Fig 1F gel/Fig 1F GAHDH.jpg]

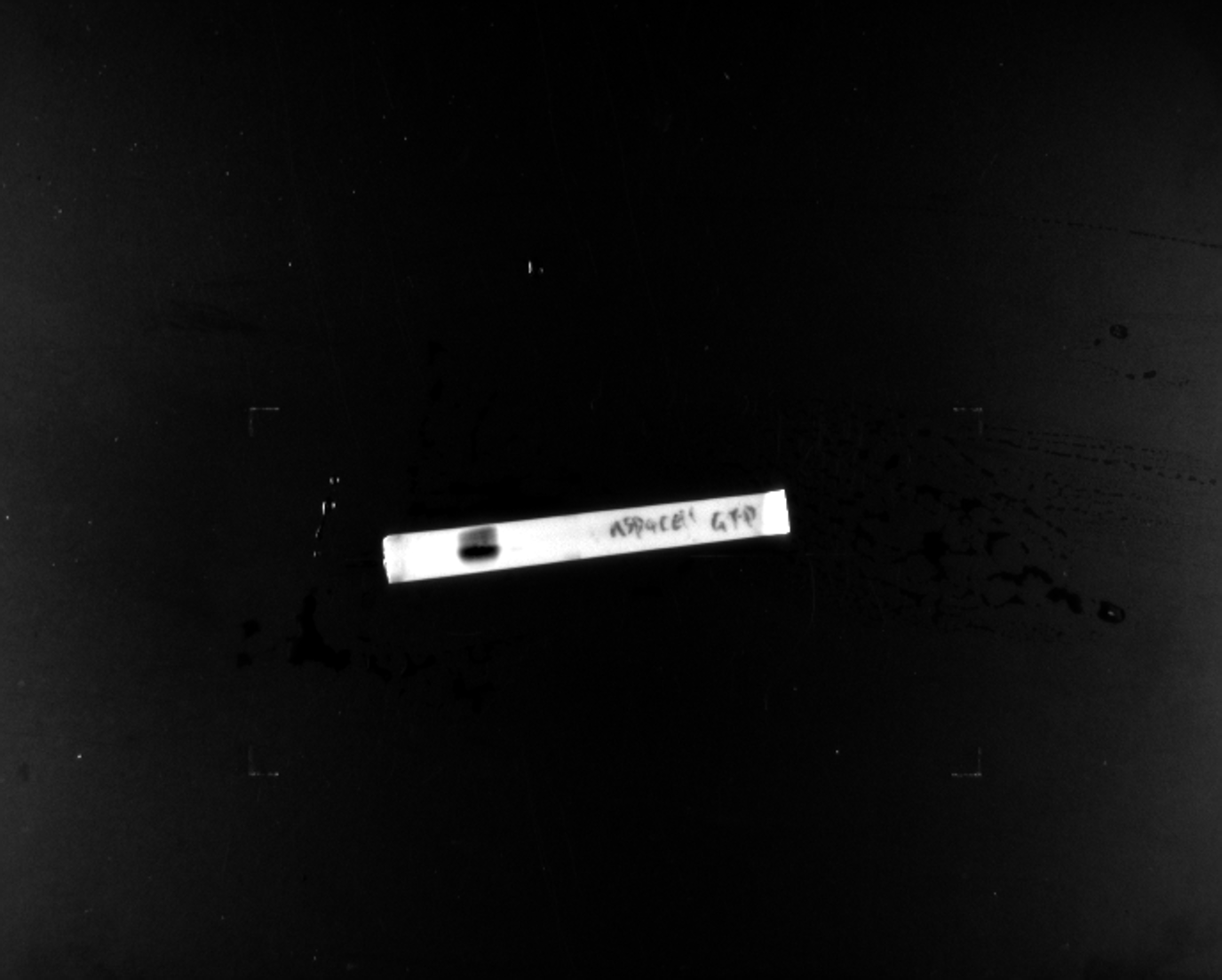

Supplement: Supplementary file 1 [file vetsci-12-00052-s001.zip › Gel原始图片/Fig 1F gel/Fig 1F GFP.Tif]

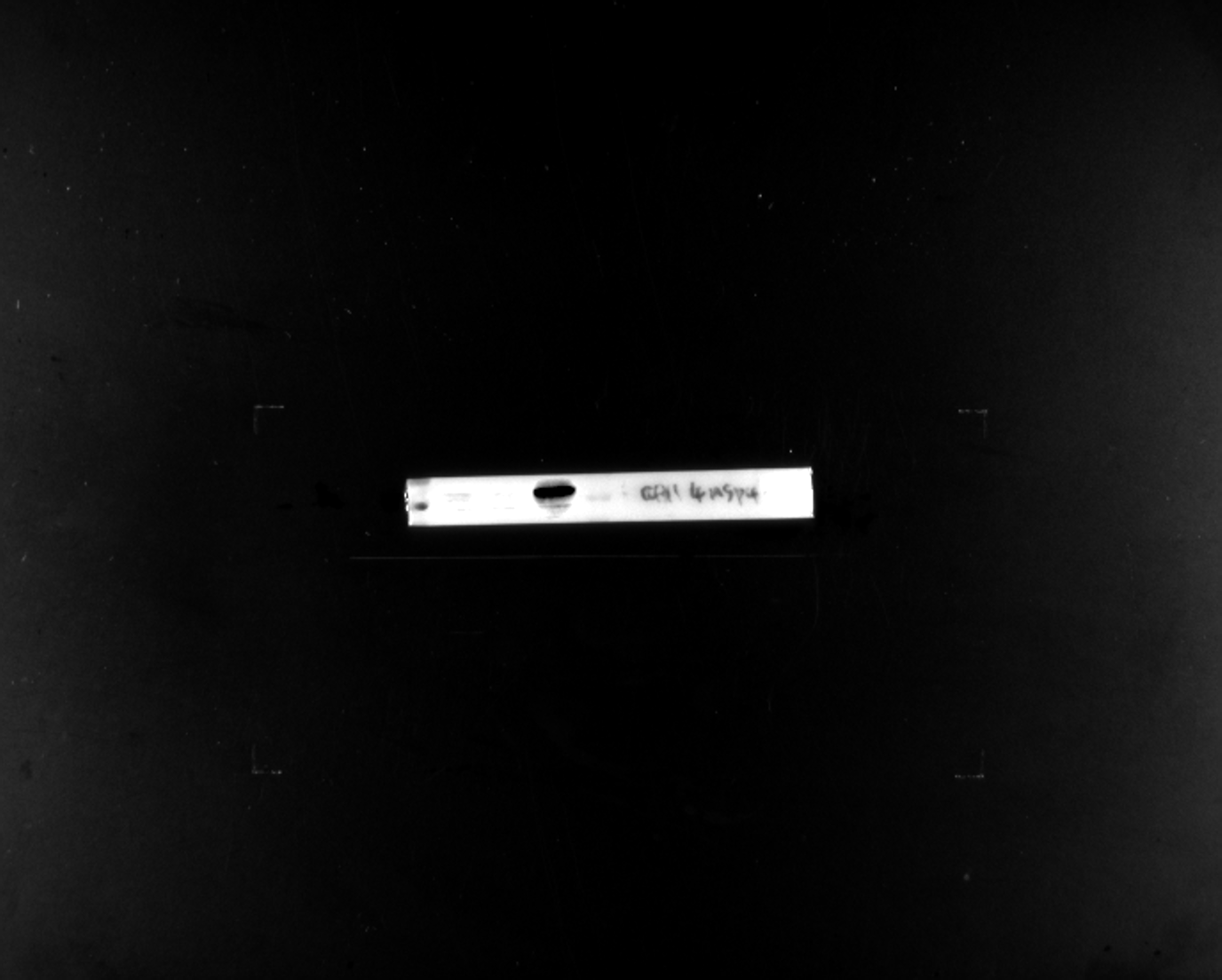

Supplement: Supplementary file 1 [file vetsci-12-00052-s001.zip › Gel原始图片/Fig 1F gel/Fig 1F Nsp4.Tif]

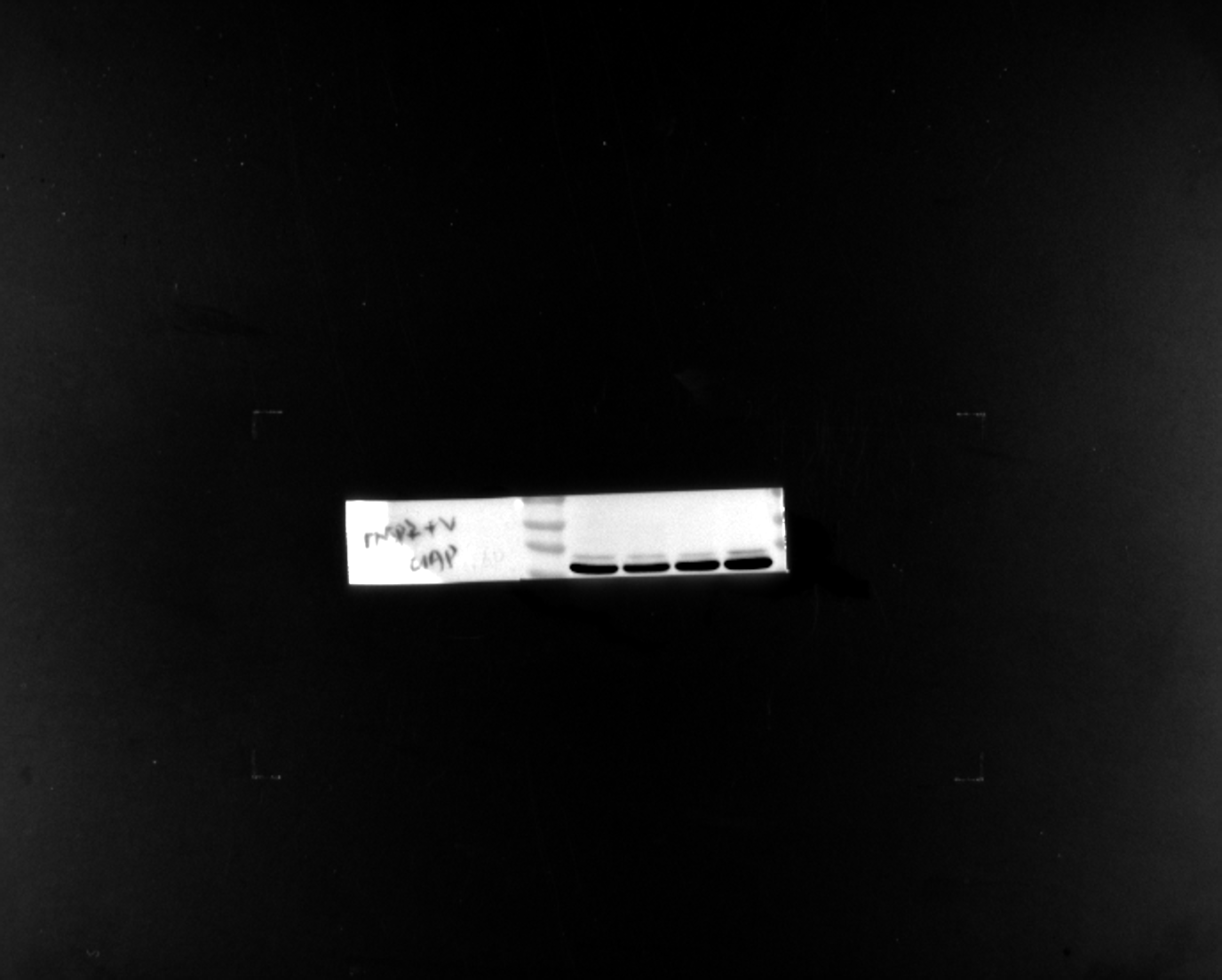

Supplement: Supplementary file 1 [file vetsci-12-00052-s001.zip › Gel原始图片/Fig 3A gel/Fig 3A GAPDH.Tif]

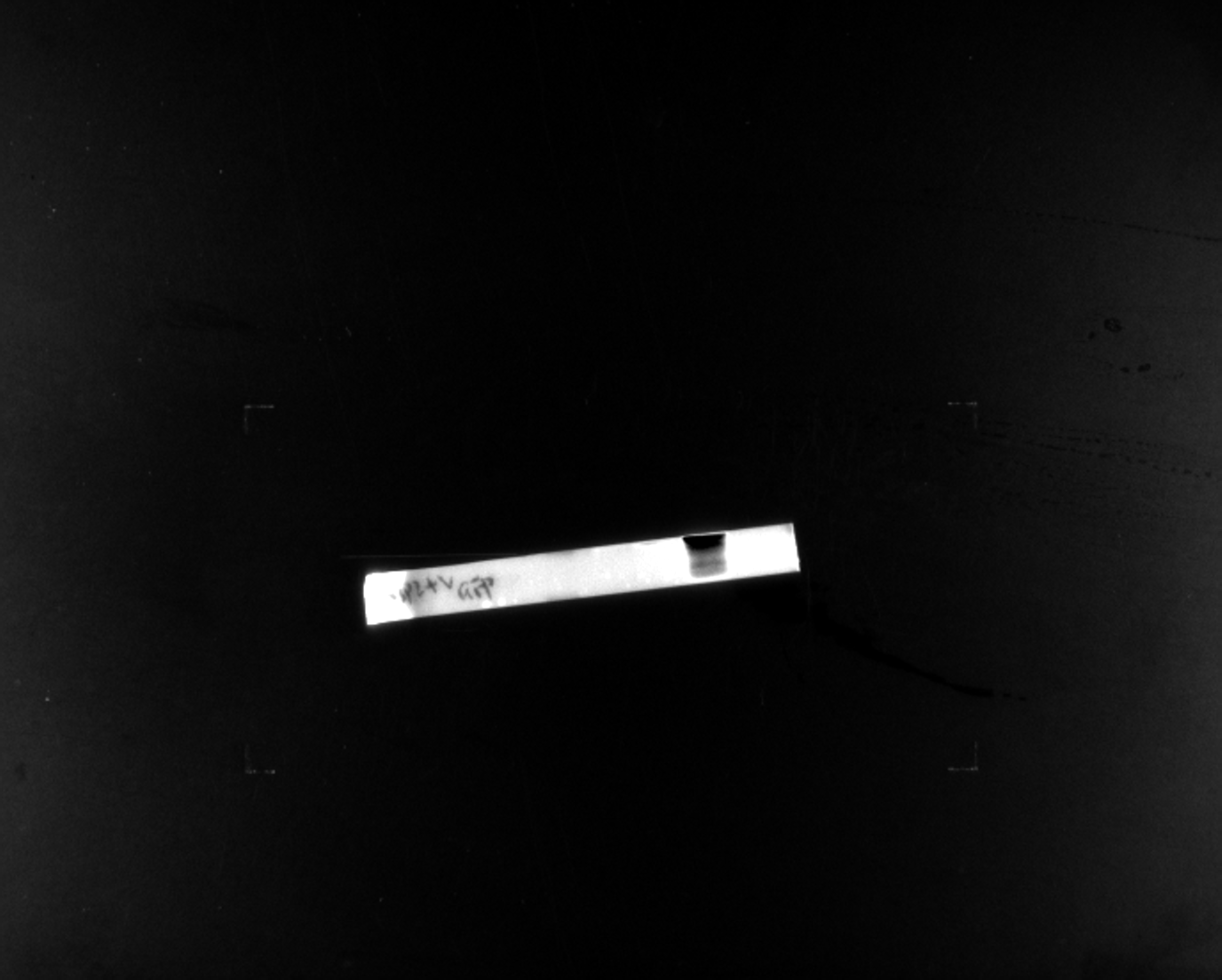

Supplement: Supplementary file 1 [file vetsci-12-00052-s001.zip › Gel原始图片/Fig 3A gel/Fig 3A GFP.Tif]

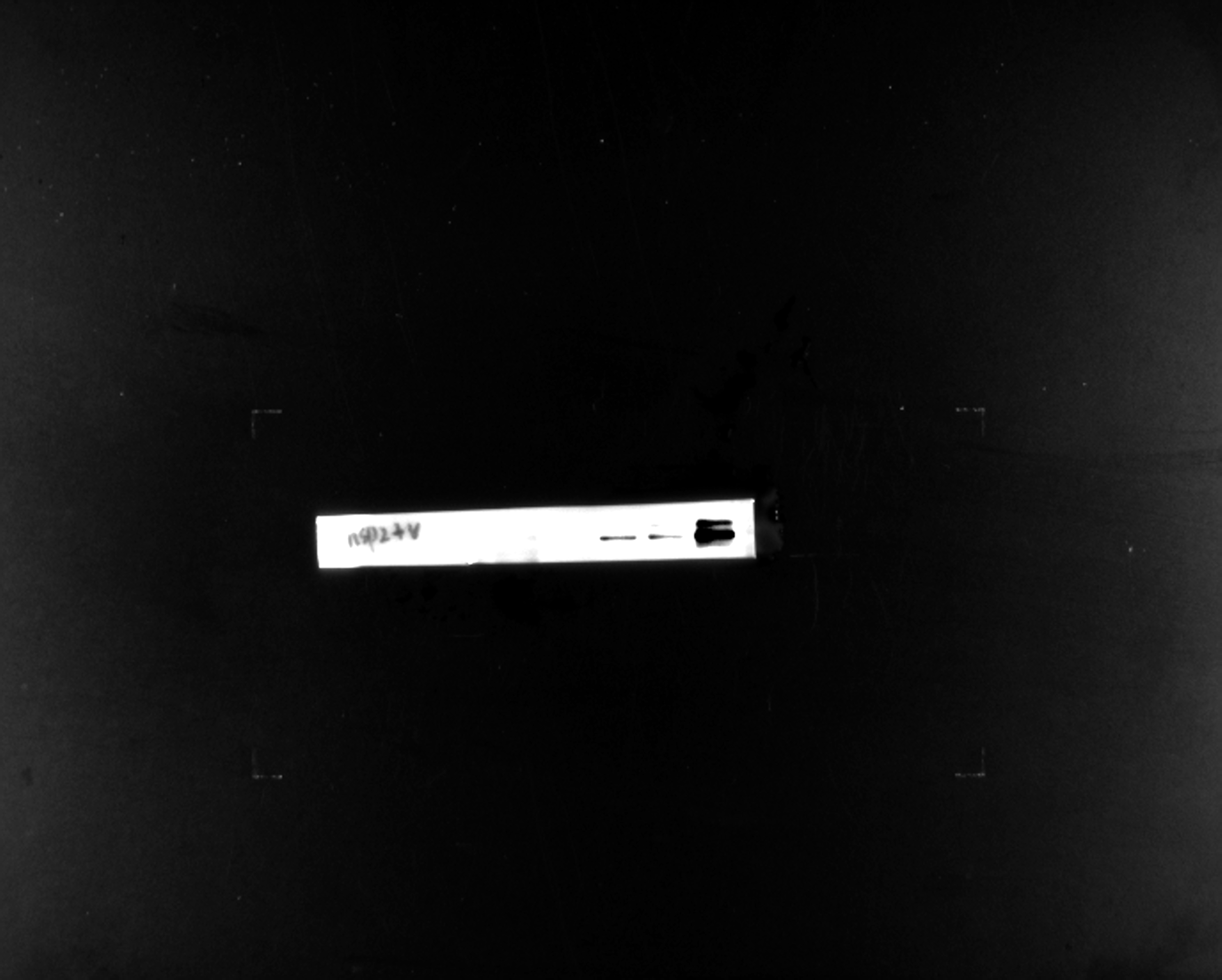

Supplement: Supplementary file 1 [file vetsci-12-00052-s001.zip › Gel原始图片/Fig 3A gel/Fig 3A Nsp2.tif]

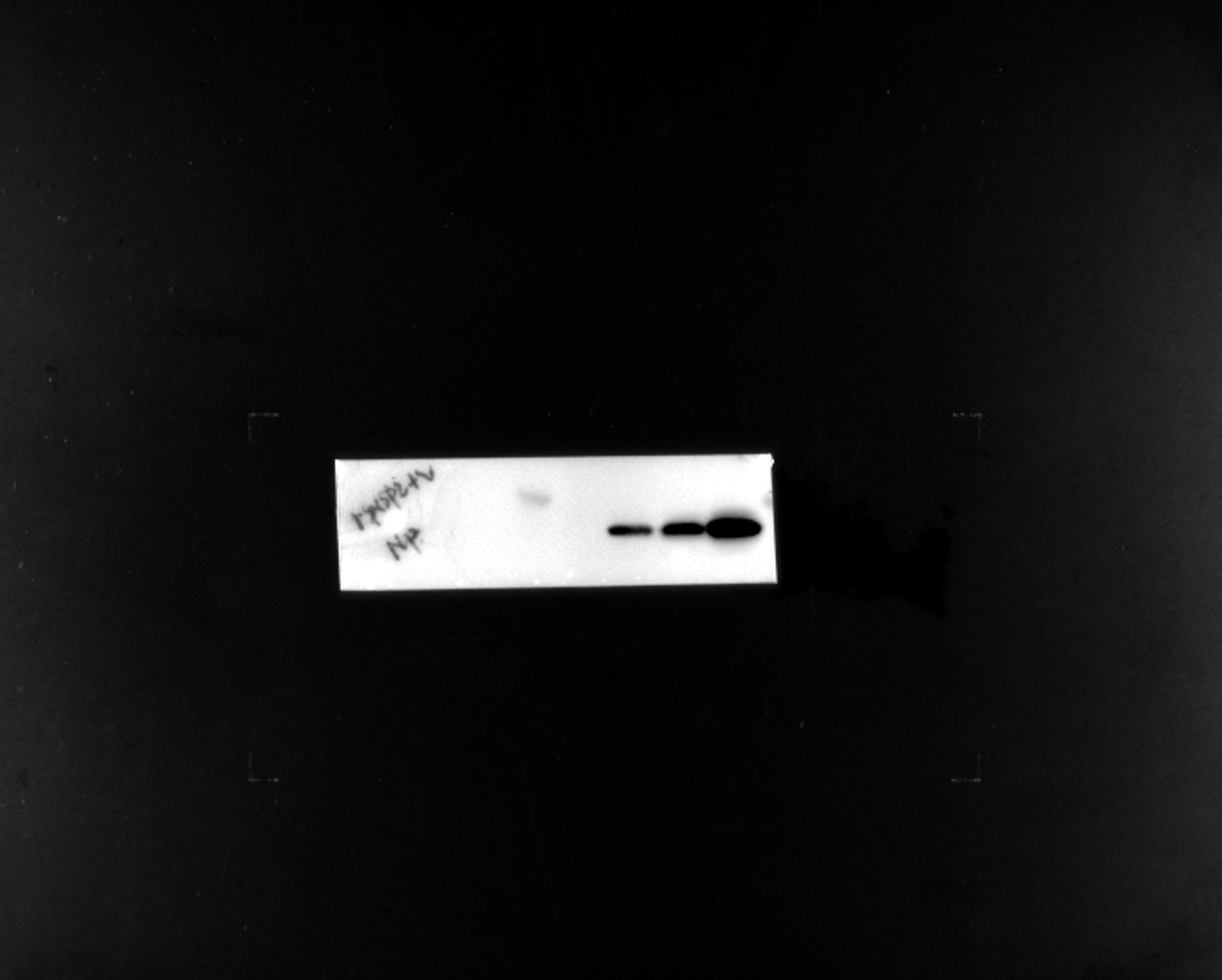

Supplement: Supplementary file 1 [file vetsci-12-00052-s001.zip › Gel原始图片/Fig 3A gel/Fig 3A PRRSV N protein.Tif]

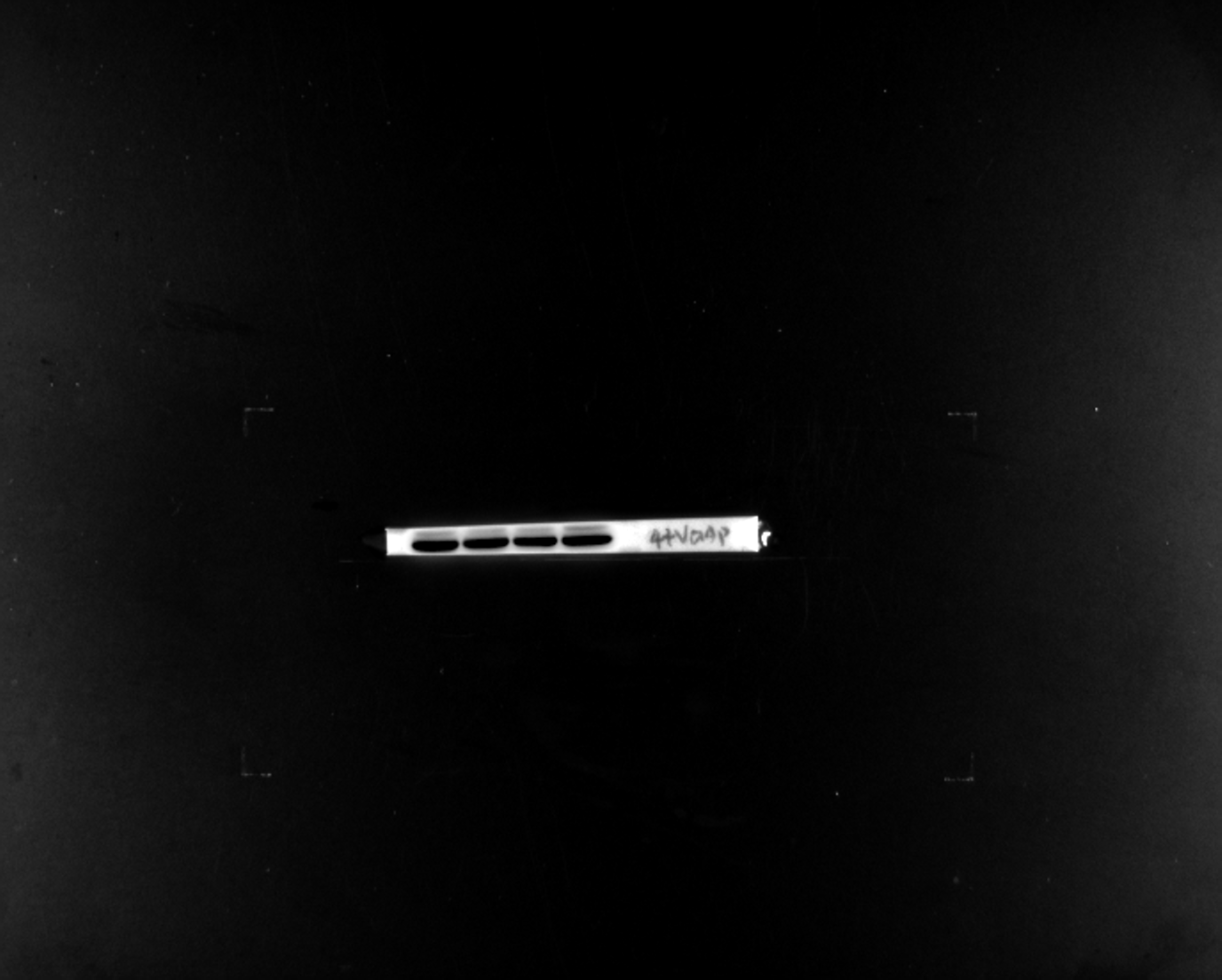

Supplement: Supplementary file 1 [file vetsci-12-00052-s001.zip › Gel原始图片/Fig 3B gel/Fig 3B GAPDH.Tif]

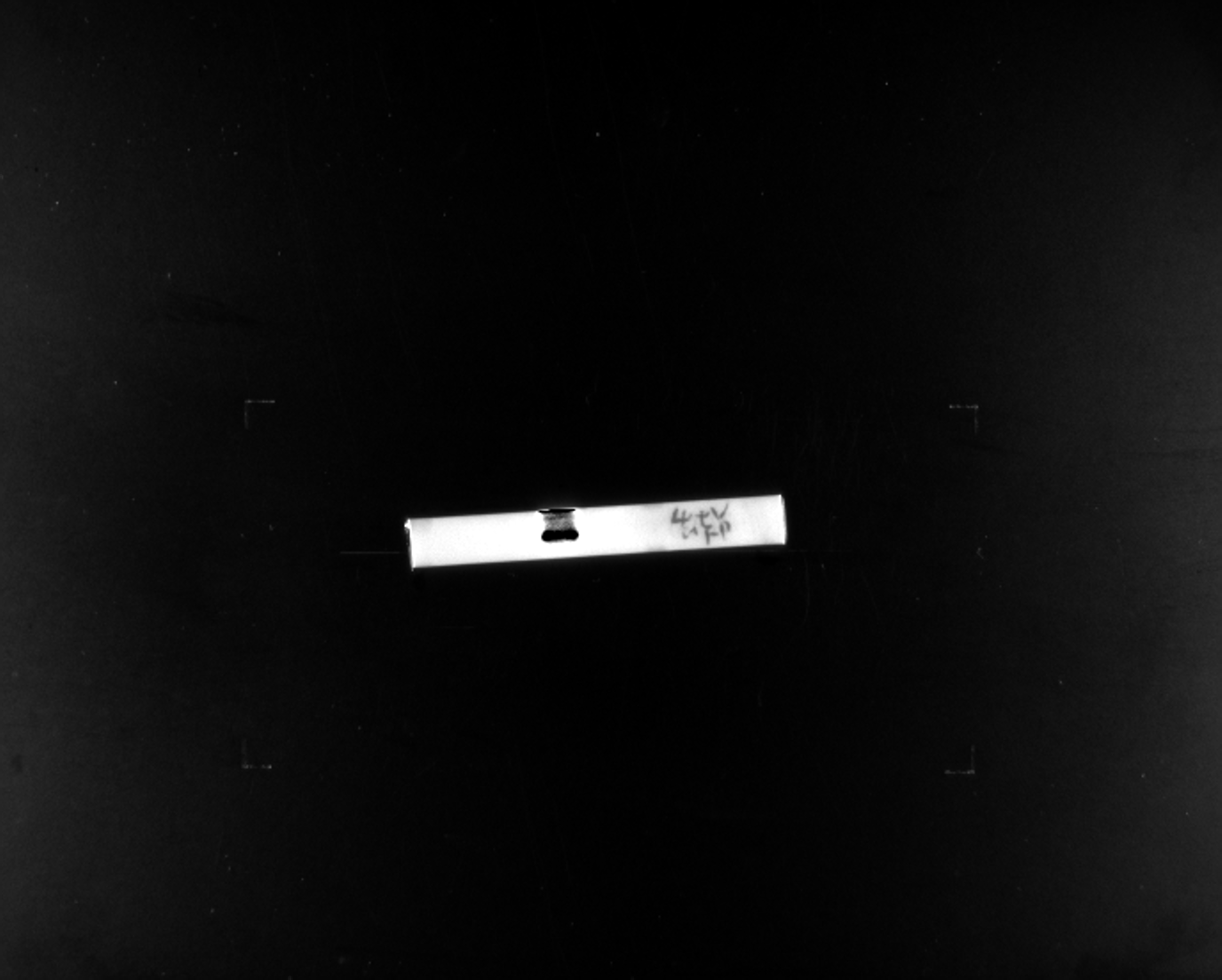

Supplement: Supplementary file 1 [file vetsci-12-00052-s001.zip › Gel原始图片/Fig 3B gel/Fig 3B GFP.Tif]

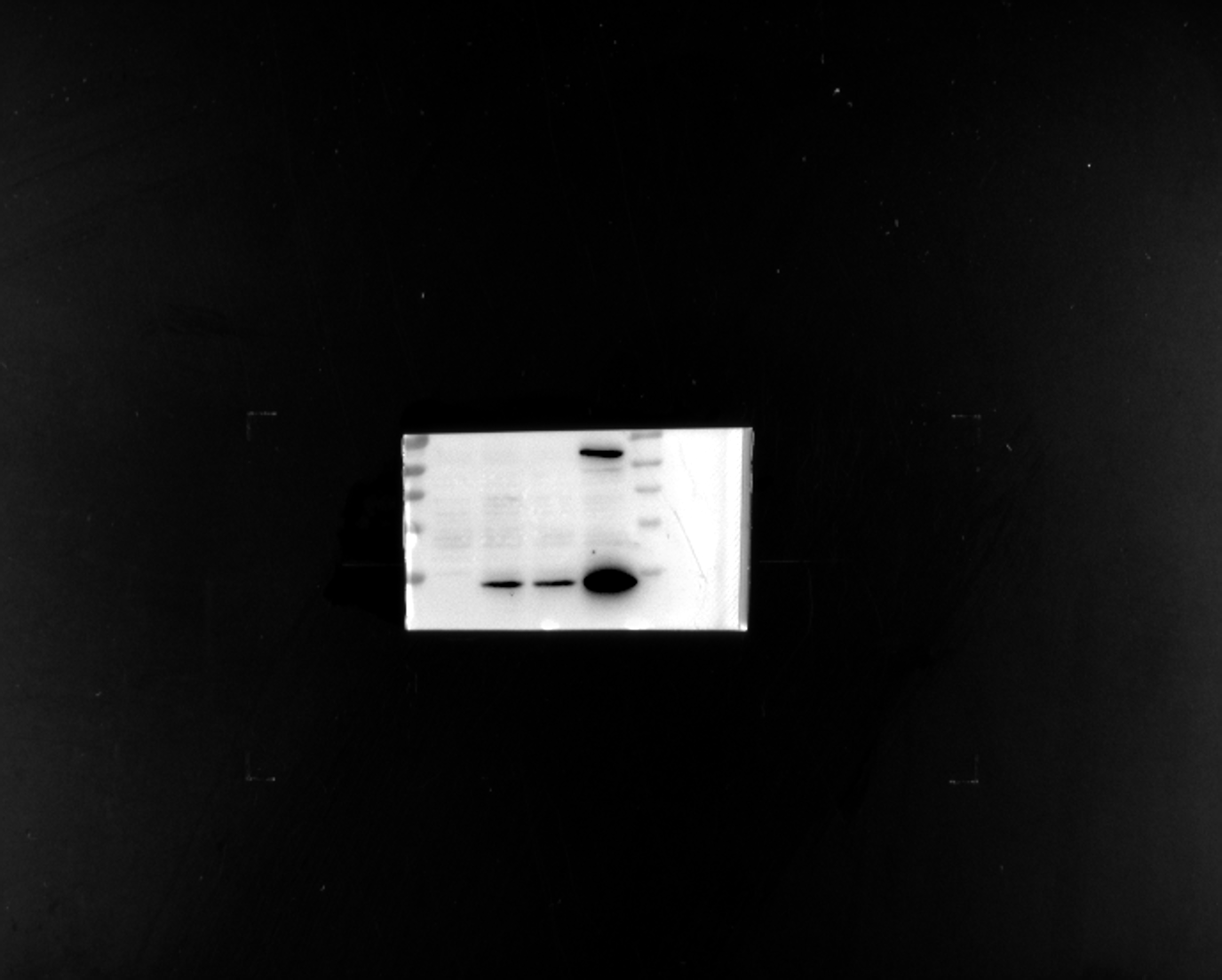

Supplement: Supplementary file 1 [file vetsci-12-00052-s001.zip › Gel原始图片/Fig 3B gel/Fig 3B Nsp4.Tif]

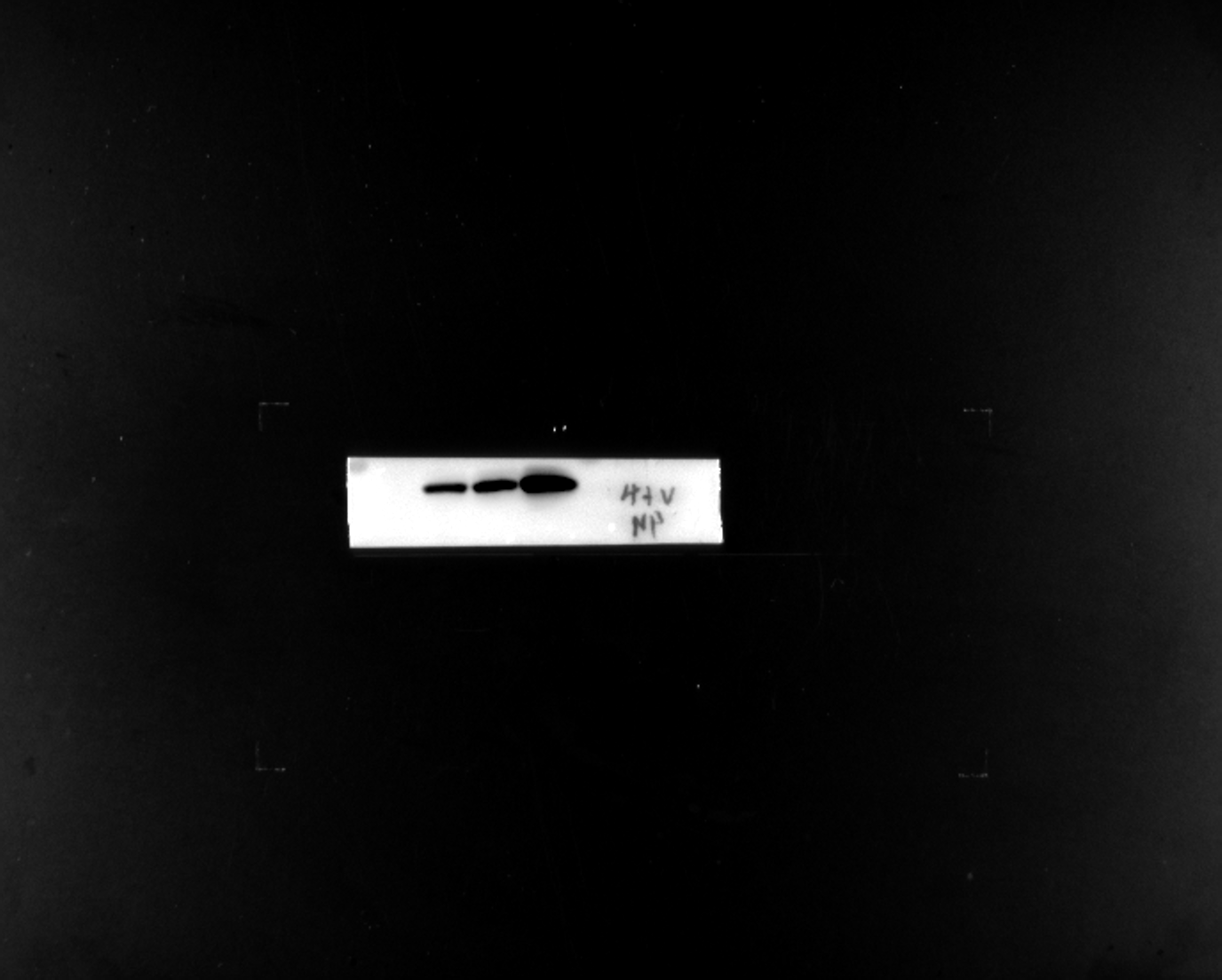

Supplement: Supplementary file 1 [file vetsci-12-00052-s001.zip › Gel原始图片/Fig 3B gel/Fig 3B PRRSV N protein.Tif]

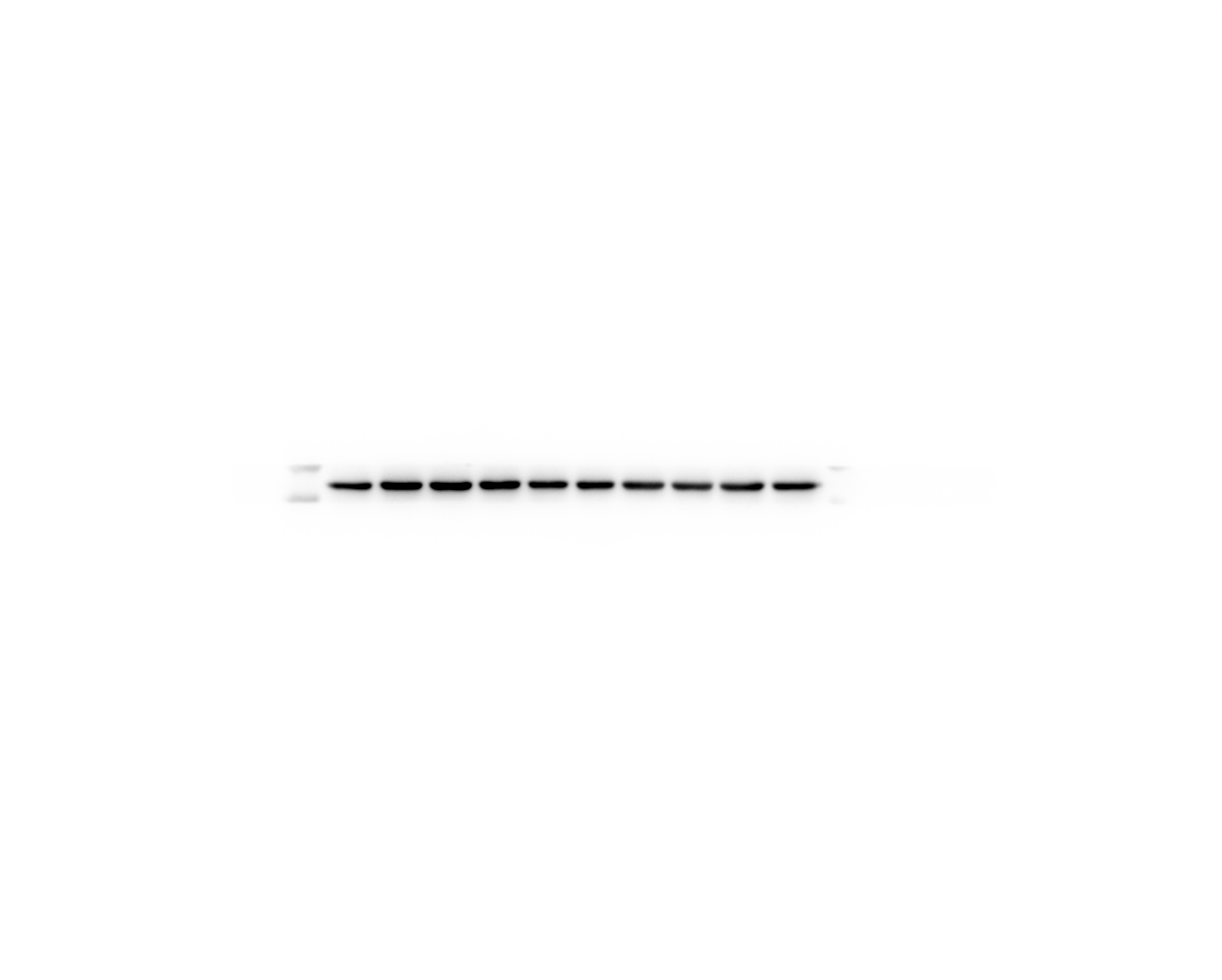

Supplement: Supplementary file 1 [file vetsci-12-00052-s001.zip › Gel原始图片/Fig 3D gel/GAPDH.Tif]

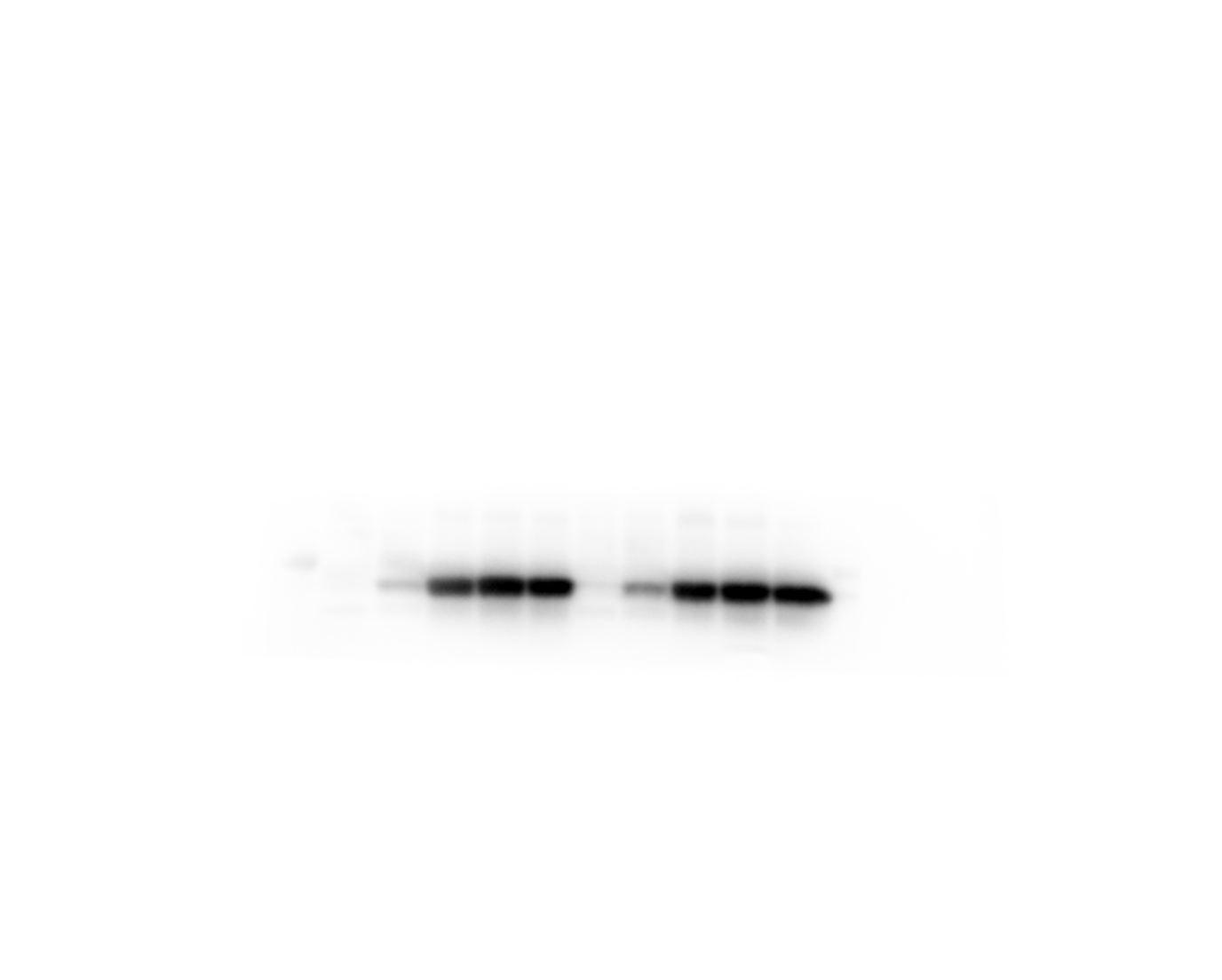

Supplement: Supplementary file 1 [file vetsci-12-00052-s001.zip › Gel原始图片/Fig 3D gel/NP.Tif]

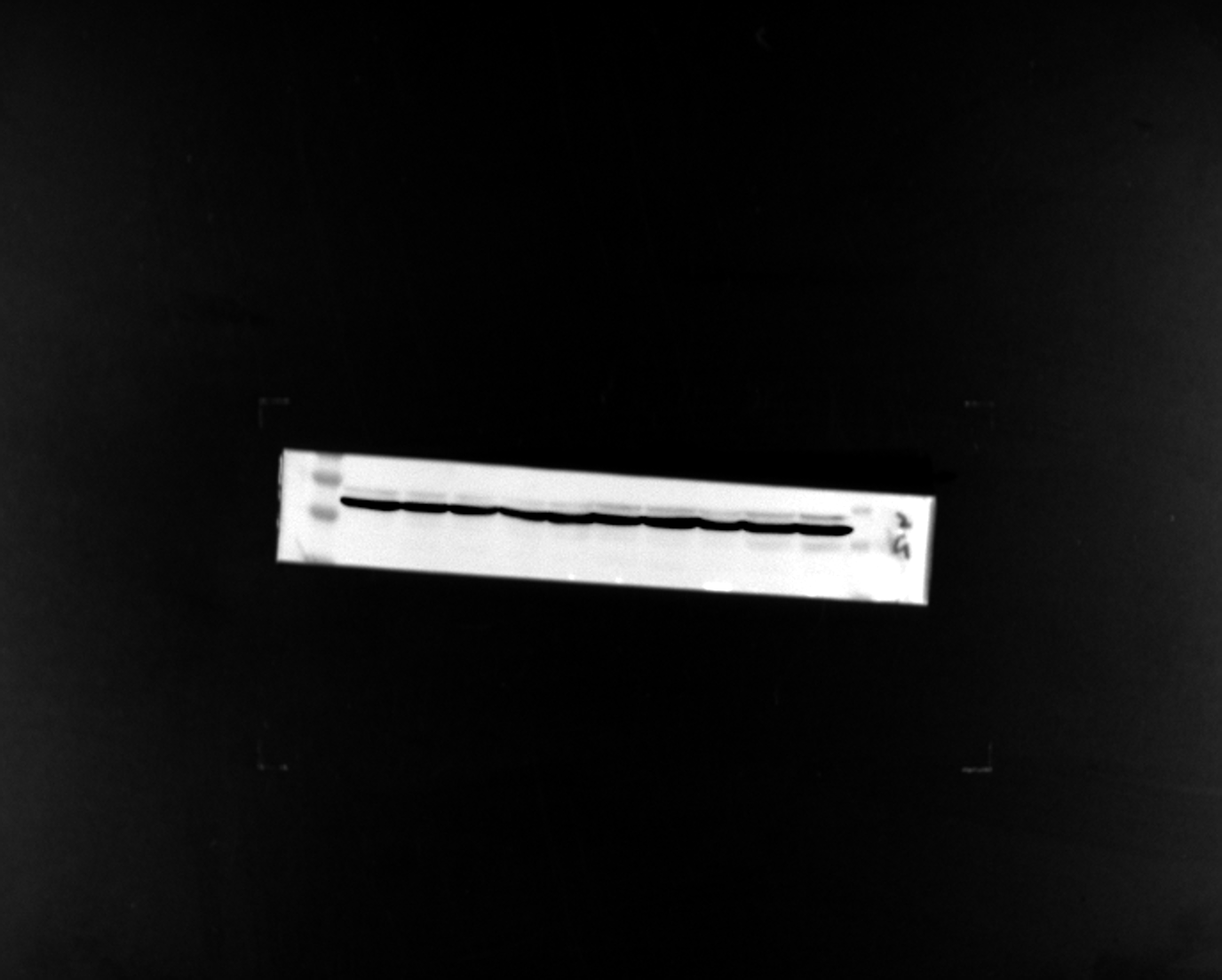

Supplement: Supplementary file 1 [file vetsci-12-00052-s001.zip › Gel原始图片/Fig3E gel/Fig 3D Western blot analysis of viral N protein levels in infected rNsp2-Marc-145 cells at different time points (GAPDH).Tif]

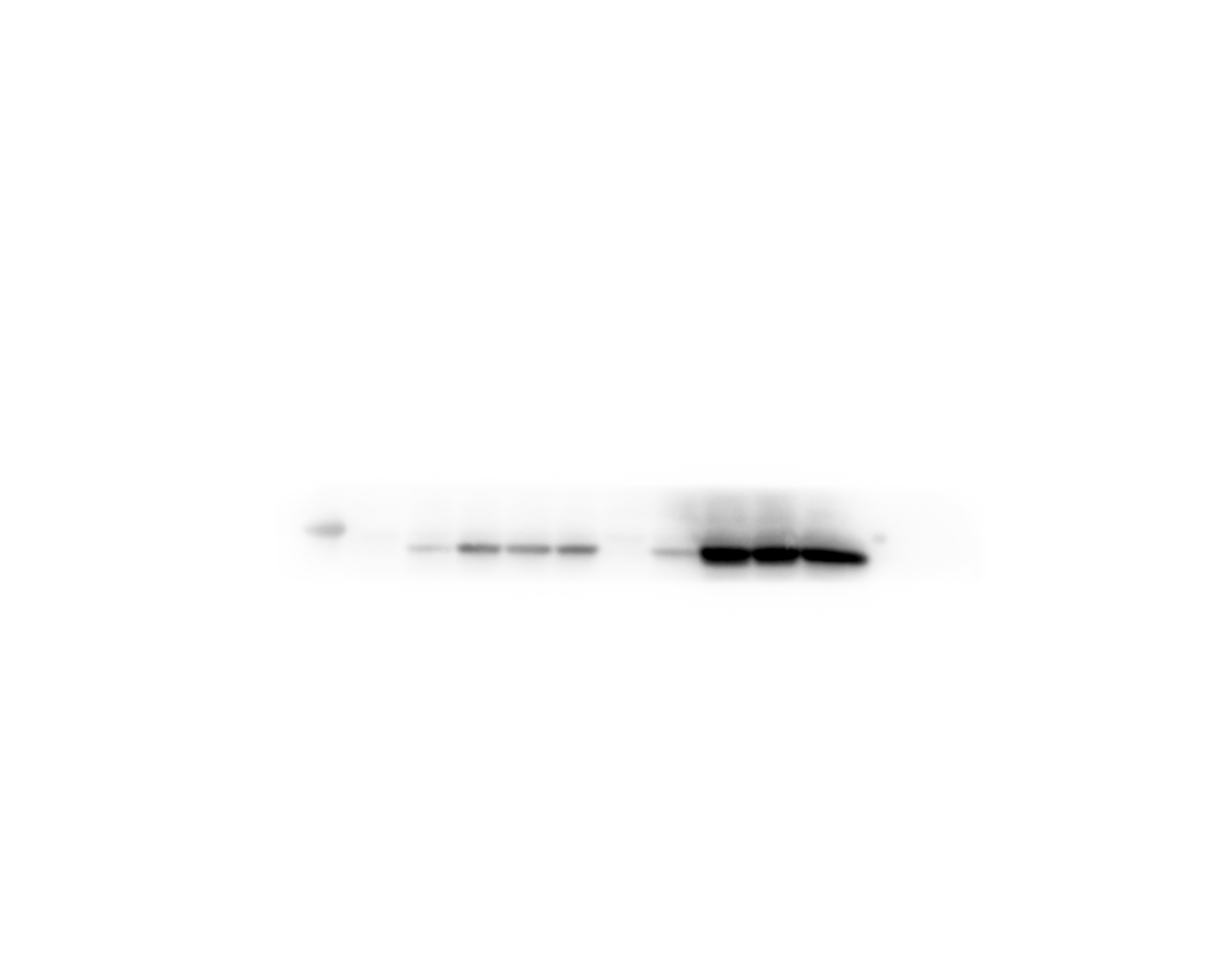

Supplement: Supplementary file 1 [file vetsci-12-00052-s001.zip › Gel原始图片/Fig3E gel/Fig 3D Western blot analysis of viral N protein levels in infected rNsp2-Marc-145 cells at different time points.Tif]

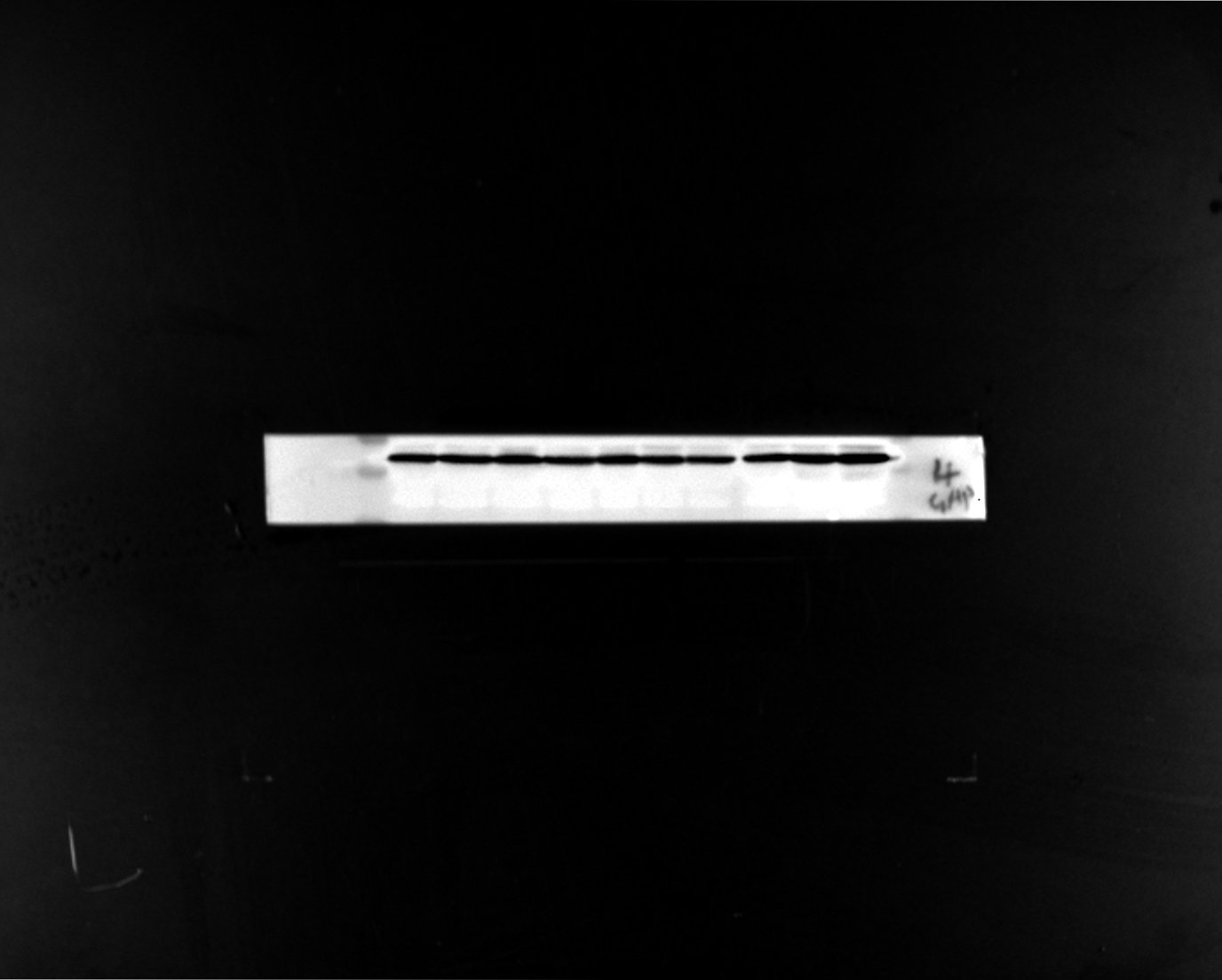

Supplement: Supplementary file 1 [file vetsci-12-00052-s001.zip › Gel原始图片/Fig3F gel/Fig 3E Western blot analysis of viral N protein levels in infected rNsp4-Marc-145 cells at different time points (GAPDH).jpg]

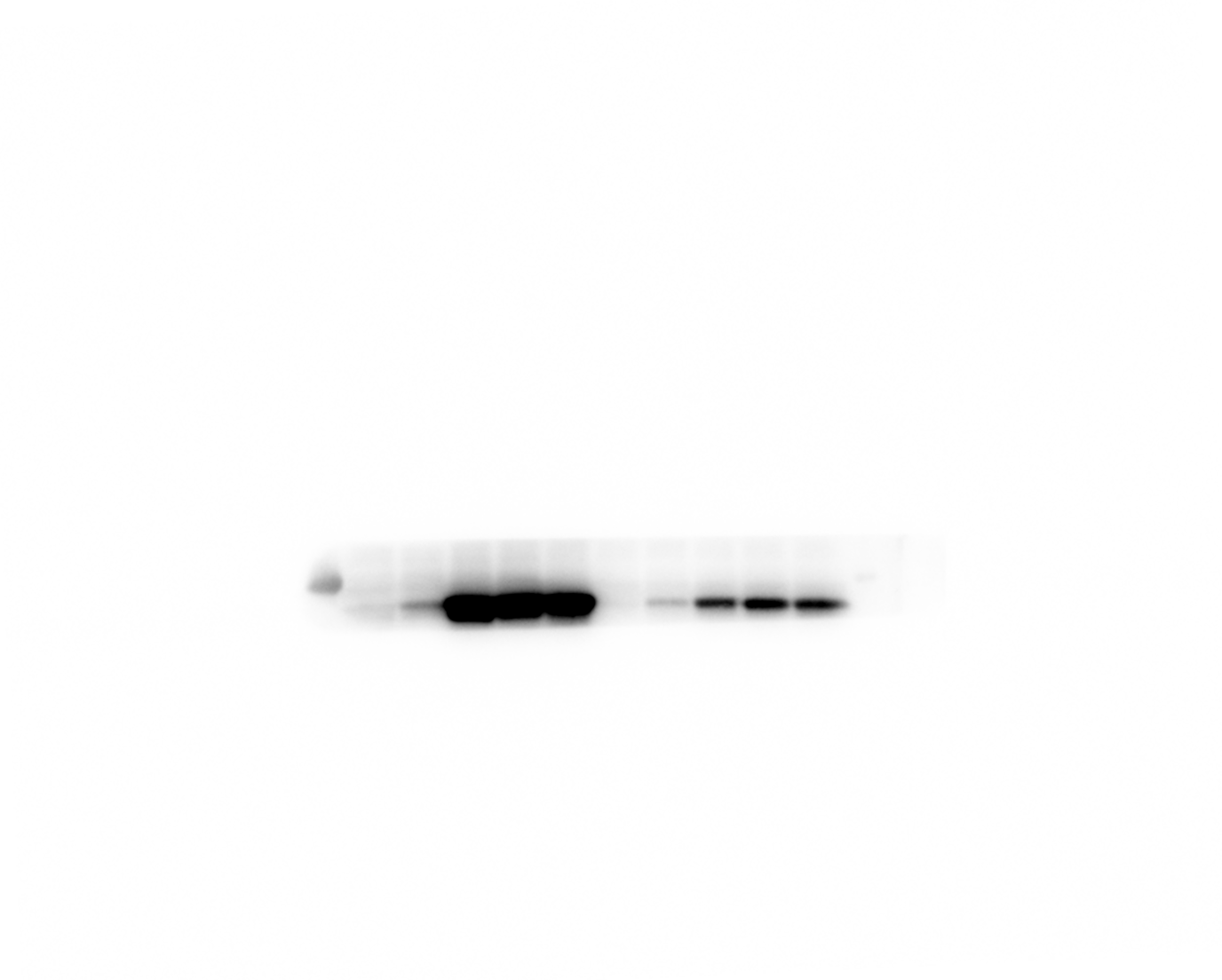

Supplement: Supplementary file 1 [file vetsci-12-00052-s001.zip › Gel原始图片/Fig3F gel/Fig 3E Western blot analysis of viral N protein levels in infected rNsp4-Marc-145 cells at different time points.Tif]
